# Health outcomes and medical response during a military deployment in Iraq: a prospective observational study of morbidity, treatments, and evacuations

**DOI:** 10.1590/1516-3180.2025.3543.25022026

**Published:** 2026-05-22

**Authors:** David Ramirez Avellaneda, Marta Elena Losa Iglesias, Ricardo Becerro de Bengoa Vallejo, Juan Gómez-Salgado, Daniel López-López, Carmen de Labra

**Affiliations:** I Department of Health Sciences, Faculty of Nursing and Podiatry, Universidade da Coruña, Ferrol (A Coruña), Spain. Research, Health and Podiatry Group. Military in active duty.; II Faculty of Health Sciences, Universidad Rey Juan Carlos, Madrid, Spain.; III Faculty of Nursing, Physiotherapy and Podiatry, Universidad Complutense de Madrid, Madrid, Spain.; IV Department of Sociology, Social Work and Public Health, Faculty of Labour Sciences, University of Huelva, Huelva, Spain; Safety and Health Postgraduate Programme, Universidad Espíritu Santo, Guayaquil, Ecuador.; V Department of Health Sciences, Faculty of Nursing and Podiatry, Universidade da Coruña, Industrial Campus of Ferrol, Ferrol (A Coruña), Spain. Research, Health and Podiatry Group.; VI Department of Physiotherapy, Medicine and Biomedical Sciences, Faculty of Nursing and Podiatry, Universidade da Coruña, Industrial Campus of Ferrol, Ferrol (A Coruña), Spain. Research, Health and Podiatry Group.

**Keywords:** Military personnel, Military medicine, Morbidity, Patient evacuation, Iraq, Military deployment, Role 1, Disease and non-battle injury (DNBI), Medical support, Forward surgical team, Telemedicine

## Abstract

**BACKGROUND:**

Overseas military deployment poses a significant healthcare challenge, requiring the prevention of morbidity associated with physical strain and the environment, as well as the provision of effective medical care to ensure operational continuity in operations.

**OBJECTIVES:**

To describe and analyze the medical care activity during the deployment of the Spanish contingent from the General Command of Ceuta at the Union III base (Baghdad) and to evaluate the recorded morbidity, applied treatments, and the need for medical evacuation.

**STUDY DESIGN AND SETTINGS:**

A prospective, observational, and analytical study was conducted during the deployment between May and November 2025 at the Union III military base in Baghdad.

**METHODS:**

All medical encounters recorded by the deployed medical services were included. Demographic and clinical variables as well as the applied treatment, need for follow-up, and evacuation, were analyzed. Statistical analysis included descriptive statistics and association tests, with a **p** value < 0.05 considered significant.

**RESULTS:**

In total, 506 medical encounters were recorded. Traumatic injuries were the most frequent reason for consultation (25.7%), followed by infectious (20.9%) and digestive (17.2%) conditions. Most cases were resolved at Role 1 level, with a low evacuation rate (3.4%) and a high degree of local resolution.

**CONCLUSIONS:**

The deployed medical support demonstrated a high resolutive capacity, with a predominance of non-combat-related pathologies and a low need for evacuation. These results highlight the importance of maintaining effective medical structures and reinforcing preventive measures to reduce morbidity during future deployments.

## INTRODUCTION

 Overseas military deployments are an essential component of the preparation, availability, and operational effectiveness of the armed forces, with purposes beyond a mere deterrent presence. In Iraq, under the umbrella of the NATO Mission Iraq (NMI), a non-combatant multinational mission aimed at advising and strengthening Iraqi defense and security institutions, Spanish personnel deployed at the Union III base (Green Zone, Baghdad) perform functions such as facility security, guard duties, escorts, personnel protection, allied training, and logistical support in the theater of operations.^ 1,2^ These operational tasks require maintenance of unit cohesion and a tiered medical support system that guarantees triage, initial treatment, and evacuation according to the allied doctrine of Roles 1 or 2 and evacuation pathways.^3^

 The Baghdad setting, characterized by high temperatures, low humidity, suspended dust, and sustained physical loads (carrying equipment, prolonged guard duties, transfers between bases, escorts, and shooting exercises), imposes significant physiological demands. Scientific evidence places acclimatization, hydration, and workload control as pillars to mitigate heat illness and preserve force performance.^4,5^ Additionally, specific preparation for load carriage is associated with a reduction in overuse morbidity and the risk of musculoskeletal injuries.^5^

 Furthermore, in the context of life on base, frequent transitions between air-conditioned interiors and hot, dusty exteriors favor the appearance of respiratory infections and certain skin conditions, potentially impacting operational continuity. In recent missions in Afghanistan and Iraq, it has been documented that disease and non-battle injuries (DNBI) account for a substantial portion of healthcare demand and represent a relevant proportion of medical evacuations with high resolution in the first echelon.^6,7^

 Beyond their operational and healthcare dimensions, military deployments involve substantial economic investments and rigorous logistical planning. According to NATO data, in 2024, military expenditure in allied countries amounted to nearly $ 1,506 million, representing an average of 2.2% of the Alliance’s GDP; meanwhile, Spain allocated approximately 1.24% of its GDP to defense spending that year (~17,2 million €).^8,9^ This highlights that operations depend not only on personnel and operational resources, but also on demanding and efficient budgetary management. 

 Literature from theaters of operations reinforces this framework. Recent studies have demonstrated that in austere environments, traumatic injuries can be managed locally with simple devices and clear referral/evacuation criteria;^10^ likewise, endemic infectious pathologies in deployed personnel, such as mucosal leishmaniasis, require protocols activatable from the area and continuity of care,^11^ while telemedicine programs offer 24/7 expert support and improve MEDEVAC/CASEVAC decision-making.^12^ Similarly, the casuistry of forward surgical teams (Role 2 forward) provides intervention thresholds for extremity injuries in comparable scenarios.^13^

 Prospective real-time morbidity data from Role 1 medical activities in allied deployments are scarce, making this study one of the few structured analyses of morbidity patterns and evacuation requirements in a desert operational environment. 

 Therefore, the objective of this study was to describe and analyze all medical attendances recorded during the deployment of the Spanish contingent at the Union III base (Baghdad) (May 26November 26, 2025), evaluating the frequency of diagnostic categories; the applied treatments and their degree of local resolution; the proportion and reasons for evacuation; and the associations between type of condition, unit profile, and rank. 

 While the existing literature provides a solid framework for DNBI patterns and medical support in combat zones, there is a need for detailed, prospective data from contemporary, non-combat advisory missions set in urban environments, such as Baghdad’s Green Zone. Such settings present unique challenges in which physiological strain and base-living conditions are the primary risk factors, unlike high-intensity combat or remote austere outposts. 

## METHODS

### Design and participants

 This study was conducted according to the STROBE criteria (STROBE Statement–Checklist of items that should be included in Observational Studies in Epidemiology) and was approved by the Ethics Committee on Medicinal Products of the Central Defense Hospital Gómez Ulla (CEIm-HCDGU). 

 This prospective, observational, and analytical study was conducted during the deployment of the Spanish contingent from the General Command of Ceuta at Union III (Green Zone, Baghdad) between May 26 and November 26, 2025. All medical encounter attendances recorded by the deployed medical service for the personnel of the said contingent were consecutively included, applying a non-probabilistic convenience sampling that exclusively covered the attended population from the Ceuta contingent. The participating units were: Legión, Regulares, Caballería, Artillería, Cuartel General, Ingenieros, and Unidad Logística No. 23 (ULOG23). Information was collected by the medical team attached to the base security missions, escort/support for training, and logistical sustainment, guaranteeing immediate care and systematic documentation of each healthcare event. 

### Data collection and definitions

 Inclusion criteria: All Spanish military personnel of the Ceuta contingent who required medical attention in operations and whose cases were recorded by the deployed medical services. The exclusion criteria were attendances corresponding to personnel not belonging to the Ceuta contingent, or incomplete records without formal documentation of care.

**The following variables were recorded:**
- Attendance number (unique identifier). Rank (enlisted, non-commissioned officer, or officer).
- Unit of origin.
- Sex (male/female).
- Diagnostic area (Traumatology, Infectious, Digestive, Dermatology, Allergology, Dentistry, Neurology, Ophthalmology, ENT, Podiatry, Urology, Cardiology, Pulmonology, Nephrology).
- Diagnosis (clinical description).
- Applied treatment.
- Follow-up (yes/no) after the first assessment.
- Evacuation (yes/no) and referral destination when applicable.

Data collection was prospective and used standardized forms completed by medical personnel during the mission. For analytical purposes, each encounter was treated as an independent observation, in line with established approaches to morbidity surveillance within military epidemiology. Subsequently, the records were digitized for analysis. 

 The study received a favorable evaluation from CEIm-HCDGU (registration no. 67/25). The data were obtained from an operational healthcare database and were treated anonymously and confidentially, without personal identification information, in accordance with the General Data Protection Regulation and Organic Law No. 3/2018 on Personal Data Protection and the guarantee of digital rights. 

### Statistical analysis

 Data were processed using SPSS version 28 (IBM Corp., Armonk, New York). Absolute and relative frequencies (%) were calculated for all the variables. Inferential analysis was performed using Pearson’s chi-square test to assess the associations between categorical variables. All tests were two-tailed, and statistical significance was set at p < 0.05. 

## RESULTS

 During the study period, 506 medical attendances were recorded, corresponding exclusively to the military personnel of the Ceuta contingent deployed at the Union III base (Baghdad). Each record was considered as an independent event; therefore, the same service member could generate more than one healthcare episode during the study period. Most attendances were men (482, 95.3%), while 24 (4.7%) were women. The distributions by rank and unit are presented in **Table 1**. 

**Table 1:** General characteristics of the sample: sex, rank, and unit of origin; n and %

**Variable**	**Category**	**n**	**%**
Sex	Male	482	95.3
	Female	24	4.7
Military rank	Enlisted	310	61.3
	Non-Commissioned Officer	92	18.2
	Officer	104	20.6
Unit of origin	Regulares	153	30.2
	Legión	132	26.1
	Cuartel General	103	20.4
	Caballería	81	16
	ULOG/ULOG23	22	4.3
	Artillería	14	2.8
	Ingenieros	1	0.2
**Total attendances**	-	**506**	**100**

**Figure 1:**
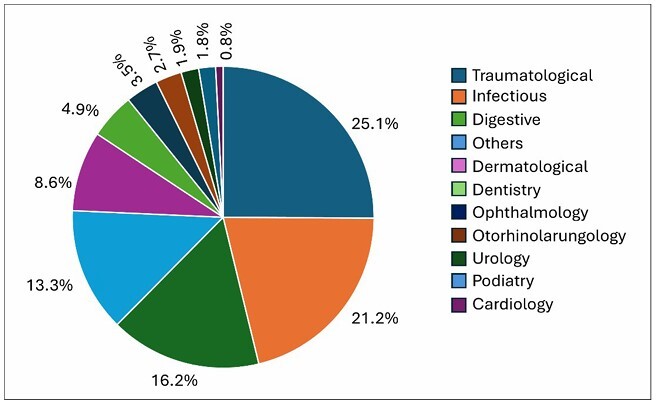
Percentage distribution of attendances by diagnostic area.

 Traumatic conditions constituted the most frequent reason for consultation, with 130 cases (25.7%), followed by infectious (106, 20.9%) and digestive (87, 17.2%) conditions. This was followed by dermatological diseases (n = 45, 8.9%) and allergological conditions (n = 29, 5.7%). Other areas with lower incidences were dentistry (26, 5.1%), neurology (23, 4.5%), ophthalmology (19, 3.8%), otorhinolaryngology (14, 2.8%), podiatry (11, 2.2%), urology (10, 2%), and cardiology (4, 0.8%), as well as isolated pulmonology and nephrology (1 case each, 0.2%). The global distribution by diagnostic area is summarized in **Table 2** and represented graphically in **Figure 1**. 

**Table 2:** Distribution of attendances by diagnostic area; n and %

**Diagnostic Area**	**n**	**%**
Traumatology	130	25.7
Infectious	106	20.9
Digestive	87	17.2
Dermatology	45	8.9
Allergology	29	5.7
Dentistry	26	5.1
Neurology	23	4.5
Ophthalmology	19	3.8
Otorhinolaryngology	14	2.8
Podiatry	11	2.2
Urology	10	2
Cardiology	4	0.8
Pulmonology	1	0.2
Nephrology	1	0.2
**Total**	**506**	**100**

**Figure 2:**
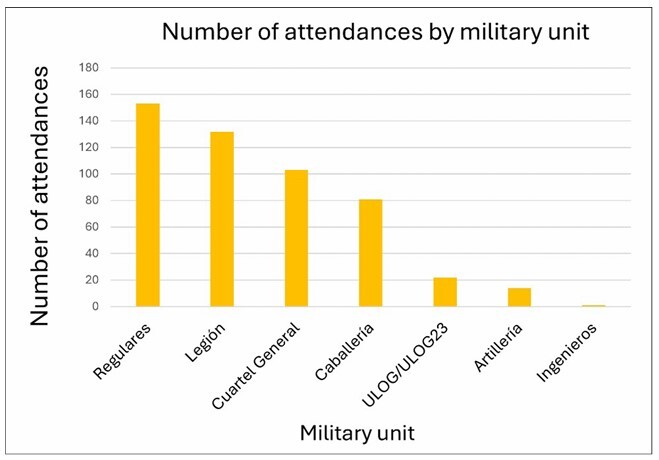
Number of attendances by military unit.

 Regarding origin, most attendances were from Regulares (153 cases, 30.2%), followed by Legión (132 cases, 26.1%). The next was Cuartel General with 103 cases (20.4%), while Caballería registered 81 attendances (16.0%). Logistic support units (ULOG/ULOG23) included 22 cases (4.3%), and Artillería included 14 cases (2.8%). Finally, a single case corresponding to Ingenieros was recorded (0.2%). This distribution reflects the operational weight of each unit and the intensity of its tasks during deployment in Baghdad (**Figure 2**). 

 Inferential analysis showed that the relationship between unit of origin and type of condition was statistically significant (χ^2^ = 277.66; df = 182; *p* < 0.001), indicating that morbidity patterns varied relevantly among the different units of the contingent. Units with a higher physical load and direct participation in security and escorting tasks (mainly Regulares, Legión, and Caballería) presented a higher proportion of traumatic injuries, whereas units with a more administrative or support component, such as Cuartel General and ULOG23, showed a broader distribution of digestive, infectious, and dermatological pathologies. Artillería and Ingenieros, with a lower number of cases, reflected more heterogeneous healthcare profiles, but with less epidemiological weight in the overall contingent. 

 Regarding military rank, most attendances corresponded to enlisted personnel, with 310 cases (61.3%), followed by officers with 104 cases (20.6%) and non-commissioned officers with 92 cases (18.2%). Inferential analysis did not show a significant association between rank and type of condition (χ^2^ = 36.46; df = 28; *p* = 0.131), indicating that enlisted personnel, non-commissioned officers, and officers presented a similar morbidity pattern, without relevant differences in the distribution of diagnostic areas based on rank. 

 Regarding clinical evolution, 99 cases (19.6%) required at least one follow-up, whereas 407 cases (80.4%) were resolved in a single medical act. Most attendances were managed entirely in the area, with 18 evacuations (3.4%) to higher echelons (reference hospitals, civilian clinics, or specialist consultations, mainly dentistry and telemedicine). Inferential analysis showed that the relationship between diagnostic area and evacuation was highly significant (χ^2^ = 96.19; df = 14; *p* < 0.001), indicating that the clinical reason clearly conditioned the need for referral. The areas that generated the highest number of evacuations were dentistry, certain traumatic conditions requiring advanced imaging tests, and some isolated cases of cardiology and respiratory pathology. In contrast, most digestive, dermatological, allergological, and mild neurological pathologies were managed completely in Role 1, without requiring MEDEVAC or external consultations. **Table 3** records exclusively the diagnostic areas that generated at least one evacuation during the study period. 

**Table 3:** Evacuations by diagnostic area (only areas with at least one evacuation are shown)

**Diagnostic area**	**Total cases**	**Evacuated**	**% Evacuation**	**Role 2E**	**Dentist**	**Telemedicine**
Traumatology	130	5	3.8%	4	0	1
Infectious	106	2	1.9%	1	0	1
Digestive	87	2	2.3%	1	0	1
Dermatology	45	1	2.2%	0	0	1
Dentistry	26	6	23.1%	0	6	0
Urology	10	1	10%	0	0	1
Cardiology	4	1	25%	1	0	0
**Total (only areas with ≥ 1 evacuation)**	**408**	**18**	**4.4%**	**7**	**6**	**5**

Note: The remaining diagnostic areas (neurology, ophthalmology, otorhinolaryngology, podiatry, pulmonology, and nephrology) recorded no evacuations during the study period.

 As is shown in **Table 4**, a total of 861 therapeutic interventions were recorded because many episodes required treatment combinations. Simple analgesics constituted the most commonly used group with 137 interventions (15.9%), followed by nonsteroidal anti-inflammatory drugs (NSAIDs), with 122 interventions (14.2%). Fluid therapy (oral or intravenous serum) was administered on 69 occasions (8%), digestive treatments and antispasmodics reached 52 interventions (6%), as did topical anti-inflammatory drugs (52, 6%). 

**Table 4:** Therapeutic interventions grouped by pharmacological families / type of measure; n and % of total interventions

**Therapeutic Category**	**n**	**%**	**Examples Included**
Simple Analgesics	137	15.9%	Paracetamol, metamizol, nolotil
Systemic NSAIDs	122	14.2%	Ibuprofen, diclofenac, naproxen, dexketoprofen
Fluid Therapy	69	8%	Oral rehydration salts, saline, ringer’s lactate, glucose
Topical anti-inflammatories	52	6%	Diclofenac gel, lidocaine patches
Digestive/antispasmodics	52	6%	Hyoscine butylbromide, omeprazole, loperamide
Systemic antibiotics	49	5.7%	Amoxicillin/clavulanate, azithromycin, ciprofloxacin
Physiotherapy/physical measures	36	4.2%	Physiotherapy, stretching, functional bandage, cryotherapy
Respiratory (mucolytics/sprays)	34	4%	Acetylcysteine, oxymetazoline
Antiseptics/local wound care	30	3.5%	Povidone-iodine, chlorhexidine
Corticosteroids (topical and systemic)	23	2.7%	Prednisone, hydrocortisone
Antihistamines	23	2.7%	Cetirizine, ebastine
Antiemetics	13	1.5%	Ondansetron, metoclopramide
Antifungals	8	0.9%	Miconazole, KETOCONAZOLE
Psychotherapy/anxiolytics	8	0.9%	Diazepam
Antivirals	4	0.5%	Acyclovir
Urological	3	0.3%	Tamsulosin
**Total**	**861**	**100%**	**-**

 Systemic antibiotics were used in 49 interventions (5.7%), mainly for respiratory, dermatological, and dental infections. Physiotherapy and physical measures (functional bandages, stretching, cryotherapy, and directed rest) comprised 36 interventions (4.2%), while respiratory treatments (mucolytics and nasal sprays) comprised 34 interventions (4%). 

 The other groups used antiseptics and local wound care less frequently (30, 3.5%), topical and systemic corticosteroids (23, 2.7%), antihistamines (23, 2.7%), antiemetics (13, 1.5%), antifungals (8, 0.9%), and psychotropic drugs (8, 0.9%). The use of antivirals was occasional (n = 4; 0.5%), and urological treatments were exceptional (n = 3; 0.3%). 

## DISCUSSION

 The results of this study revealed a morbidity pattern typical of military deployments in desert environments with high operational loads, dominated by musculoskeletal injuries, respiratory infections, and digestive disorders. This profile is consistent with that described in previous operations in Iraq and Afghanistan, where DNBI represent 60–70% of the total healthcare demand and constitute the main cause of loss of operational capacity.^14,15^ Similar morbidity distributions have also been reported in contemporary deployments in Lebanon (UNIFIL), Mali (EUTM), and the wider Sahel region, where non-combat conditions, environmental exposure, and sustained operational tempo continue to drive DNBI as the principal source of healthcare utilization.^13,15 ,26^

 In our series, traumatic injuries represented 25.7% of the total attendances, which is consistent with previous studies indicating that patrols, prolonged guard duties, sustained physical load, and continuous use of protective equipment increase the risk of overuse musculoskeletal injuries in deployments in hot zones.^16,17^ Although this proportion is lower than that described in highintensity maneuvers, where it can exceed 45–50%,^18^ it is expected that in a more static scenario such as Union III, overload syndromes, low back pain, and mild sprains predominate compared to acute trauma associated with intense training. This distribution coincides with the musculoskeletal injury patterns described by Knapik et al.^19^ and Jones et al.,^16^ who relate accumulated fatigue and prolonged effort to a higher incidence of lumbar pain and lower extremity injuries. These findings reinforce the need for targeted musculoskeletal injury-prevention programs, including load-management strategies, pre-deployment conditioning, and routine physiotherapy availability, to mitigate cumulative fatigue effects in Role 1 environments.^5,16,17,19^

 Infectious diseases accounted for 20.9% of consultations, mostly corresponding to upper respiratory tract infections. This proportion is comparable to that described in previous deployments in Iraq and Afghanistan, where respiratory illnesses constitute one of the main causes of DNBI, representing between one-fifth and one-fourth of the healthcare demand in operations. Factors such as abrupt thermal changes between air-conditioned environments and extremely hot exteriors, coexistence in closed spaces, and continuous exposure to suspended dust have been identified as key predisposing elements for this type of pathology.^20^ This pattern has also been documented in series from the Armed Forces Health Surveillance Division (AFHSD), which attributes a key role to thermal contrast and mucosal dryness in predisposing to uncomplicated pharyngitis and bronchitis.^21^

 Digestive disorders, the third most frequent category (17.2%), stood out above the usual rate of exercise in the national territory, which is consistent with the operational environment of Baghdad. Previous studies have shown that changes in dietary habits, variable water chlorination, thermal stress, and dehydration predispose personnel to gastroenteritis, abdominal cramps, and functional constipation in deployed personnel.^22,23^ Similarly, dermatological alterations (8.9%) were frequent in dry climates, where sweat retained by the continuous use of equipment and friction favored the appearance of irritative dermatitis, mycoses, and folliculitis. 

 One of the most relevant findings was the statistically significant difference between military units and diagnostic areas. Units more exposed to physical and security tasks – Regulares, Legión, and Caballería – had the highest proportion of traumatic cases, whereas Cuartel General and Logistic Unit (ULOG) presented a more diversified spectrum, with a predominance of digestive, respiratory, and dermatological pathologies. This pattern is consistent with the literature describing a higher burden of DNBI in combat units than in support units in relation to physical intensity, mission type, and environmental exposure during deployment.^14^

 In contrast, no significant differences were observed by military rank, which coincides with previous studies indicating that in prolonged operational deployments, health risk is determined mainly by the function performed and operational exposure rather than by hierarchical rank.^20^

 Regarding evacuation, a significant association was identified between the diagnostic area and the need for referral. Evacuations to Role 2E, to the American clinic in Union III, or via telemedicine from Hospital Gómez Ulla were mainly concentrated in dentistry, traumatology, and cardiology, which usually require complementary tests that are not available in Role 1 or specialist evaluation. The evacuation rate in the present study, well below 10%, is consistent with previous literature, where more than 85–90% of DNBI cases are resolved at the first healthcare echelon.^24,25^ This high degree of local resolution reflects the adequate capacity of the deployed medical team and the effectiveness of triage according to the allied doctrine. 

 The analysis of treatments showed a typical Role 1 care pattern, with the wide use of nonsteroidal anti-inflammatory drugs, simple analgesics, fluid therapy, antibiotics for respiratory or skin infections, and frequent use of physiotherapy and functional measures. The high proportion of combined interventions evidences the clinical variability of cases and the need for multimodal approaches, something already described in previous operations where mixed symptoms (pain + overload + dehydration + mild infections) are common.^26^ Together, these results mirror trends observed in contemporary deployments in the Sahel, Lebanon, and Eastern Europe, enhancing the broader contextual relevance of the findings.^13,15 ,26^

 This study has several operational implications. First, it reinforces the importance of injury prevention programs for overload, hydration control in hot environments, and adequate acclimatization, all of which are fundamental pillars to decreasing morbidity in desert zones.^4,5^ Second, it highlights the value of telemedicine in the theater of operations, where it is not always possible to have specialists, aligning with recent experiences that have demonstrated that teleconsultation reduces unnecessary evacuations and improves the continuity of care.^27^

 Finally, the data underscore the need to adapt healthcare resources to the actual observed morbidity profile, especially by reinforcing the capacity to manage digestive, respiratory, and dermatological pathologies, and maintaining fast evacuation pathways for dentistry, traumatology, and cardiology. 

 Overall, the results of this work confirm that most morbidities in Union III are concentrated in non-combat-related conditions, generally mild and manageable at Role 1, but have a significant operational impact if not addressed early. Adequate healthcare planning, anticipation of climate-specific risks, personnel training, and the availability of diagnostic and treatment equipment adapted to the environment constitute key elements in guaranteeing the sustained operability of the contingent. 

 This study has several limitations that should be considered. First, its observational and single-center design, while reflecting the reality of a specific deployment, limits the generalizability of the findings and prevents the establishment of causal relationships. Second, there is a potential for under-reporting of minor conditions that personnel might have self-treated or not considered severe enough to seek medical attention. Third, the study period (May to November) coincided with the hottest months in Iraq, which likely influenced the incidence of heat-related, dermatological, and certain infectious pathologies; therefore, the morbidity pattern described may not be fully representative of annual cycles. 

 Despite these limitations, the data provide a valid and detailed snapshot of healthcare demands and medical response capacity during contemporary military deployment in this theater of operation. 

## CONCLUSIONS

 The morbidity of the Spanish contingent deployed in Union III was concentrated in non-combat-related conditions, mainly traumatic, infectious, and digestive conditions, in line with those described in other deployments in desert environments. Significant differences were observed in the disease pattern according to the unit of origin, with a higher burden of musculoskeletal pathology in Regulares, Legión, and Caballería and a more diversified profile (digestive, respiratory, and dermatological) in Cuartel General and ULOG, underscoring the need to adapt preventive measures and healthcare resources to the mission type of each unit. Military rank (enlisted, non-commissioned officer, officer) was not significantly associated with the type of condition, suggesting that, in this operational scenario, risk exposure is determined mainly by function and unit rather than by rank. These findings may inform future medical planning, preventive strategies, and resource allocation aimed at optimizing Role 1 healthcare support and sustaining operational readiness for overseas military deployment. 

## Data Availability

The datasets generated during the current study are not publicly available due to confidentiality and security restrictions but are available from the corresponding author on reasonable request.
